# The Evolution of Device-Assisted Enteroscopy: From Sonde Enteroscopy to Motorized Spiral Enteroscopy

**DOI:** 10.3389/fmed.2021.792668

**Published:** 2021-12-23

**Authors:** Fredy Nehme, Hemant Goyal, Abhilash Perisetti, Benjamin Tharian, Neil Sharma, Tony C. Tham, Rajiv Chhabra

**Affiliations:** ^1^Department of Gastroenterology and Hepatology, School of Medicine, Saint Luke's Hospital, University of Missouri Kansas City (UMKC), Kansas City, MO, United States; ^2^Department of Medicine, The Wright Center for Graduate Medical Education, Scranton, PA, United States; ^3^Division of Interventional Oncology and Surgical Endoscopy (IOSE), Parkview Cancer Institute, Fort Wayne, IN, United States; ^4^Department of Gastroenterology and Hepatology, University of Arkansas for Medical Sciences, Little Rock, AR, United States; ^5^Division of Interventional Oncology and Surgical Endoscopy (IOSE), Parkview Cancer Institute, Fort Wayne, IN, United States; ^6^Department of Endoscopy, Indiana University School of Medicine, Fort Wayne, IN, United States; ^7^Division of Gastroenterology, Ulster Hospital, Belfast, United Kingdom

**Keywords:** double-balloon enteroscopy (DBE), deep enteroscopy, spiral enteroscopy, device-assisted enteroscopy, motorized enteroscopy, small bowel

## Abstract

The introduction of capsule endoscopy in 2001 opened the last “black box” of the gastrointestinal tract enabling complete visualization of the small bowel. Since then, numerous new developments in the field of deep enteroscopy have emerged expanding the diagnostic and therapeutic armamentarium against small bowel diseases. The ability to achieve total enteroscopy and visualize the entire small bowel remains the holy grail in enteroscopy. Our journey in the small bowel started historically with sonde type enteroscopy and ropeway enteroscopy. Currently, double-balloon enteroscopy, single-balloon enteroscopy, and spiral enteroscopy are available in clinical practice. Recently, a novel motorized enteroscope has been described with the potential to shorten procedure time and allow for total enteroscopy in one session. In this review, we will present an overview of the currently available techniques, indications, diagnostic yield, and complications of device-assisted enteroscopy.

## Introduction

Up until the end of the 20^th^ century, the available options for small bowel evaluation were limited owing to the length of the small intestine and its anatomy. Push enteroscopy, the main technique, had a limited insertion depth and diagnostic yield (1). Intraoperative enteroscopy allowed complete small bowel evaluation but was associated with a high morbidity and mortality approaching 17 and 5% respectively (2). Capsule endoscopy was first reported in 2001 opening up the small bowel for diagnostic approaches, but was not able to close the gap in therapeutic interventions (3) (Table 1). The introduction of double-balloon enteroscopy (DBE) in 2001 enabled endoscopic scrutiny of the entire small bowel with intervention capabilities such as tissue sampling with biopsies, mucosal injection, polypectomy, hemostatic techniques, stricture dilation, and retrieval of foreign bodies (4). DBE remains the most studied and established deep enteroscopy (DE) technique to date. Additional methods were later introduced such as single-balloon enteroscopy (SBE) in 2007 (5) and spiral enteroscopy (SE) in 2008 (6). A novel motorized spiral enteroscope was described in 2015 allowing faster and easier progression into the small bowel (7). These techniques are known as “device-assisted enteroscopy” (DAE). DAE is a generic term for assisted progression of the enteroscope into the small bowel. Assistance is provided by overtubes, balloon catheters, or other stiffening devices (8–10).

**Table 1:** Diagnostic yield of video capsule endoscopy for various indications.

**Indication**	**Diagnostic yield (%)**
Obscure gastrointestinal bleeding	44
Acute gastrointestinal bleeding	64–87
Abdominal pain	3–21
NSAID enteropathy	5–60
Crohn's disease	39–50
Celiac disease	54
Familial adenomatous polyps	29
Peutz-Jeghers Syndrome	22–59

The field of DAE continues to evolve with the development of new enteroscopes taking therapeutic endoscopy in the small bowel to another level. Endoscopic retrograde cholangiopancreatography (ERCP) and even cholangioscopy are nowadays feasible with the help of DAE in patients with altered anatomy (11). In this review, we will highlight the latest DAE developments, the emerging clinical results, and future directions.

## Historical Device-Assisted Enteroscopy Techniques

### Sonde Type Enteroscopy

The first successful total enteroscopy was reported in 1971 using a ropeway and a sonde method. The sonde type consisted of a 5-mm forward-viewing fibroscope that can be passed transnasally and migrates distally to the stomach. It is then pushed through the pylorus with a gastroscope passed through the mouth and carried by peristalsis of a balloon inflated at the tip (12). The procedure was uncomfortable, painful, and lasted 6–8 h. It also did not allow tissue sampling, tip deflection, or therapeutic interventions. Only 50–80% of the mucosa could be visualized and up to 75% of the time, the terminal ileum could not be visualized (13).

### Ropeway Type Enteroscopy

The ropeway enteroscope consists of insertion of a long intestinal Teflon string that is advanced orally and discharged from the anus. Once this step is finished, typically requiring 24 h, the ropeway enteroscope can be pulled through the gastrointestinal tract with the aid of the string. Visualization and biopsy of the small bowel are possible, however traction on the string increases the risk of perforation and stenotic lesions disallowed the passage of the string and limited the effectiveness of this device (14, 15). The sonde and ropeway methods were cumbersome, technically challenging, time-consuming, and did not achieve wide acceptance in clinical practice. They have since been replaced by more effective deep enteroscopy techniques.

## Current Device-Assisted Enteroscopy Techniques

### Push Enteroscopy

For nearly 30 years, push enteroscopy (PE) was the preferred method and consisted of using a long endoscope with a standard diameter allowing visualization of the esophagus, stomach, duodenum, and proximal jejunum. Bleeding sources in the proximal small bowel up to 50–70 cm from the pylorus can be rapidly excluded with this method, however visualization of the entire small bowel is not possible. Compared to other DAE, PE has shorter sedation and procedure time while antegrade balloon-enteroscopy has significantly greater depth of insertion (230 vs 80 cm, *p* < 0.001) and diagnostic yield (63 vs 44%, *p* < 0.001). In addition, deep enteroscopy identifies additional lesions in deeper parts of the small bowel in most PE-positive patients (16).

### Double-Balloon Enteroscopy

The advent of video capsule endoscopy (VCE) in 2001 led to an increasing need for a reliable endoscopic method for direct access to the small bowel for histopathological confirmation or performance of endoscopic therapies. The development of DBE in 2001 resulted in a paradigm shift in diagnostic and therapeutic approaches in the small bowel. The DBE system (DBE, Fujifilm, Tokyo, Japan) comprises an enteroscope, an overtube, and a balloon-pump system with an inflatable balloon at the distal end of the enteroscope and a second balloon attached to the overtube. DBE may be performed in antegrade or retrograde manner and standard length endoscopic accessories can be used (17). After passing the duodenum or the ileo-cecal valve, the small bowel can be pleated by inflating and deflating the two balloons in tandem order leading to a much greater depth of insertion compared to push enteroscopy. This is known as a pull-and-push technique (18). There are three types of DBE available including a diagnostic, therapeutic, and a short model. The short DBE is engineered to overcome technically-challenging therapeutic ERCP procedures in patients with surgically altered anatomy.

The depth of intubation is estimated between 240 cm and 360 cm during the anterograde approach and 100–140 cm for the retrograde approach (19–21). Tee et al. found no distinct learning curve with antegrade DBE while technical success rates for retrograde DBE defined as achieving stable overtube placement in the ileum or finding the target lesion continued to increase over time during the study. The authors estimated at least 30–35 cases of retrograde DBE under supervision were needed to achieve a good technical success rate of more than 75% (22).

DBE is the most prospectively studied technique in terms of safety, diagnostic, and therapeutic yield. Total enteroscopy defined as the intubation of the entire small bowel was reported at 44% in a systematic review including 12,823 DBE procedures with an overall diagnostic yield of 68.1% (23).

Complications associated with DAE became increasingly recognized following the introduction of these new techniques. In addition to the known endoscopic complications of bleeding, perforation, and sedated associated complications, DBE has been associated with pancreatitis. Pooled minor and major adverse events in a large systematic review were 9.1% and 0.72% respectively (23).

The first reports of pancreatitis post DBE were published in 2006 (24). Several studies then reported up to 50% of patients had high levels of amylase and lipase following DBE and a few developed clinical signs of acute pancreatitis (25, 26). In large cohorts, the frequency of pancreatitis was estimated at 0.2–0.34% and the majority of the cases were reported with the antegrade route (23, 24). The pathogenesis of pancreatitis is thought to be secondary to mechanical stress on the pancreas or the papilla during the push-and-pull maneuver. One study noted a correlation between hyperamylasemia and the insertion depth and the number of pull maneuvers during DBE (27). Therefore, avoiding mechanical stress to the pancreas through slow retraction of the endoscope and the papilla by only using the balloon in deeper parts of the duodenum is recommended to reduce the risk of pancreatitis after DBE.

Bleeding after DBE has been reported particularly after interventional procedures. In a cohort of 2,362 DBE procedures, bleeding rate was 0.8% and only 0.1% after diagnostic procedures. The risk of perforation increases in those with prior abdominal surgeries. It is estimated at 0.1–0.3% in diagnostic procedures and 0.8–2.9% after small bowel polypectomy (28–30).

DAE are typically more time consuming than upper and lower gastrointestinal endoscopies and the risk of sedation-related complications should be taken into account. These complications were reported in 0.5% of cases in one database (28). Several studies have reported on the safety of DAE in the elderly (31, 32).

### Single-Balloon Enteroscopy

The single-balloon enteroscope (SBE, Olympus Medical Systems Corporation, Tokyo, Japan) consists of one balloon attached to the tip of an overtube without the balloon attached to the tip of the endoscope. This was designed to streamline the push-and-pull technique leading to shorter set-up time, and less burdensome balloon control panel (33). The main technical difference between SBE and DBE is the need to angulate the tip of the SBE before the pulling maneuver to compensate for reduced stability (34). One diagnostic and one therapeutic SBE models are available.

The depth of intubation during antegrade SBE is between 133 to 256 cm past the ligament of Treitz and 73–163 cm for retrograde SBE past the ileocecal valve. The rate of complete enteroscopy is lower than DBE between 15 to 25% while the diagnostic yield is comparable at 47 to 60% (35–38). The range of therapeutic procedures offered is similar to DBE. Overall adverse event rate is also comparable to DBE at 1% with potentially higher risk of deep submucosal tears if the endoscope tip is flexed particularly in the setting of adhesions or strictures (39). The power suction maneuver consisting of maximum suction power to hold the small intestine during the insertion of the overtube may result in less damage to the mucosa than does the hook shape (40).

### Conventional Spiral Enteroscopy

Spiral enteroscopy (Spirus Medical Inc., Stoughton, Massachusetts) was initially introduced in 2007 and consists of a manually rotatable overtube with a helical design called the Discovery Small Bowel that is positioned on a thin flexible enteroscope. The intestine is evaluated using a rotate-to-advance technology where the small bowel is retraced on the overtube with slight rotation allowing rapid advancement of the endoscope with a stable positioning. This allows meticulous examination of the small bowel on both insertion and withdrawal of the enteroscope (41). Most studies have described using spiral enteroscopy with the antegrade approach. The average depth of intubation ranges between 200 cm and 346 cm (42). Spiral enteroscopy allows reduction of total procedure time, with a similar diagnostic and therapeutic yields to DBE and a comparable depth of maximal insertion (DMI) (42, 43). The rate of total enteroscopy remains low barely approaching the 10% benchmark mainly due to difficult retrograde passage (43).

Akerman et al. reported major complication rates of 0.3%. In 2,950 patients, 8 perforations were reported with no incidence of acute pancreatitis, suggesting that SE has a lower risk of acute pancreatitis than DBE and SBE (44). Studies suggest that only about 5 procedures are required for competency in SE by an otherwise trained endoscopist (45). Conventional spiral enteroscopy is no longer available in the market since the introduction of motorized spiral enteroscopy discussed below in detail.

### Balloon-Guided Endoscopy

Balloon-guided endoscopy (NaviAid, Smart Medical Systems, Ra'anana, Israel) consists of a permanently integrated inflatable balloon at the tip of the endoscope (single-balloon) which can be used with an additional through-the-scope NaviAid AB balloon catheter through the working channel (double-balloon). The NaviAid AB balloon can also be used with a standard adult colonoscope with a 3.7 mm working channel, a principle called on-demand enteroscopy. The through-the-scope balloon catheter is advanced into the lumen and used as an anchoring device inside the small bowel to enable deep enteroscopy. Limited data reported a mean DMI of 120 cm for antegrade enteroscopy and 110 cm for retrograde enteroscopy with rapid procedure times (46, 47).

### Motorized Enteroscopy

In 2015, clinical evaluation of the first motorized version of the SE system started with the first human case of PowerSpiral Enteroscopy (PSE, Olympus Medical Systems Corporation, Tokyo, Japan) (7). PSE consists of a 168 cm long flexible endoscope that is compatible with the latest EXERA III endoscopy system. It includes a large 3.2-mm accessory channel and a separate dedicated irrigation channel. These additions reduce challenges in small bowel therapeutics and potential wear and tear on the endoscopist with less instrument exchanges. The system incorporates a user-controlled electric motor embedded in the endoscope's handle to rotate the spiral tube attached on the endoscope's insertion tube. Rotation is activated by a foot pedal switch. While the overtube pleats the bowel on the insertion tube, the resistance applied to the tissue is measured *via* a LED display to prevent bowel damage (48). This reduces the resources needed for training and personnel. With PSE withdrawal, the endoscopist should provide counterclockwise rotation to prevent the creation of shear forces and allow the small bowel to unscrew off the spiral.

In a prospective feasibility study of 140 peroral PSE procedures performed under general anesthesia, the technical success was 97% with diagnostic and therapeutic yields of 74.2% and 68.2% consecutively. The median DMI was 450 cm with a median insertion time of 25 min. Panenteroscopy to the cecum was achieved in 10.6% of the cases. The adverse event rate was 14.4% including one delayed perforation and one bleeding Malory-Weiss lesion. The risk of pancreatitis appears significantly low (49, 50).

In a study including 30 patients with indications for total enteroscopy, the total enteroscopy rate was 70.6, 16.6% with the antegrade approach alone and 53.4% with bidirectional approach (51). This rate seems to be comparable or even better than the rate of total enteroscopy in DBE of 40–60%, and much better than SBE and SE given substantial improvement in retrograde enteroscopy success rate. DMI by the retrograde approach was reported at 140 cm during a median of 35 min (52). Shortened PSE procedure time is likely due to the elimination of the push and pull reduction with balloon enteroscopy. To note, prophylactic esophageal bougie dilation has been performed in clinical studies to aid passage of the PSE through the upper esophageal sphincter but the real-world necessity of this step remains unknown.

## Conventional Indications for Device-Assisted Enteroscopy

### Bleeding

**Figure 1:**
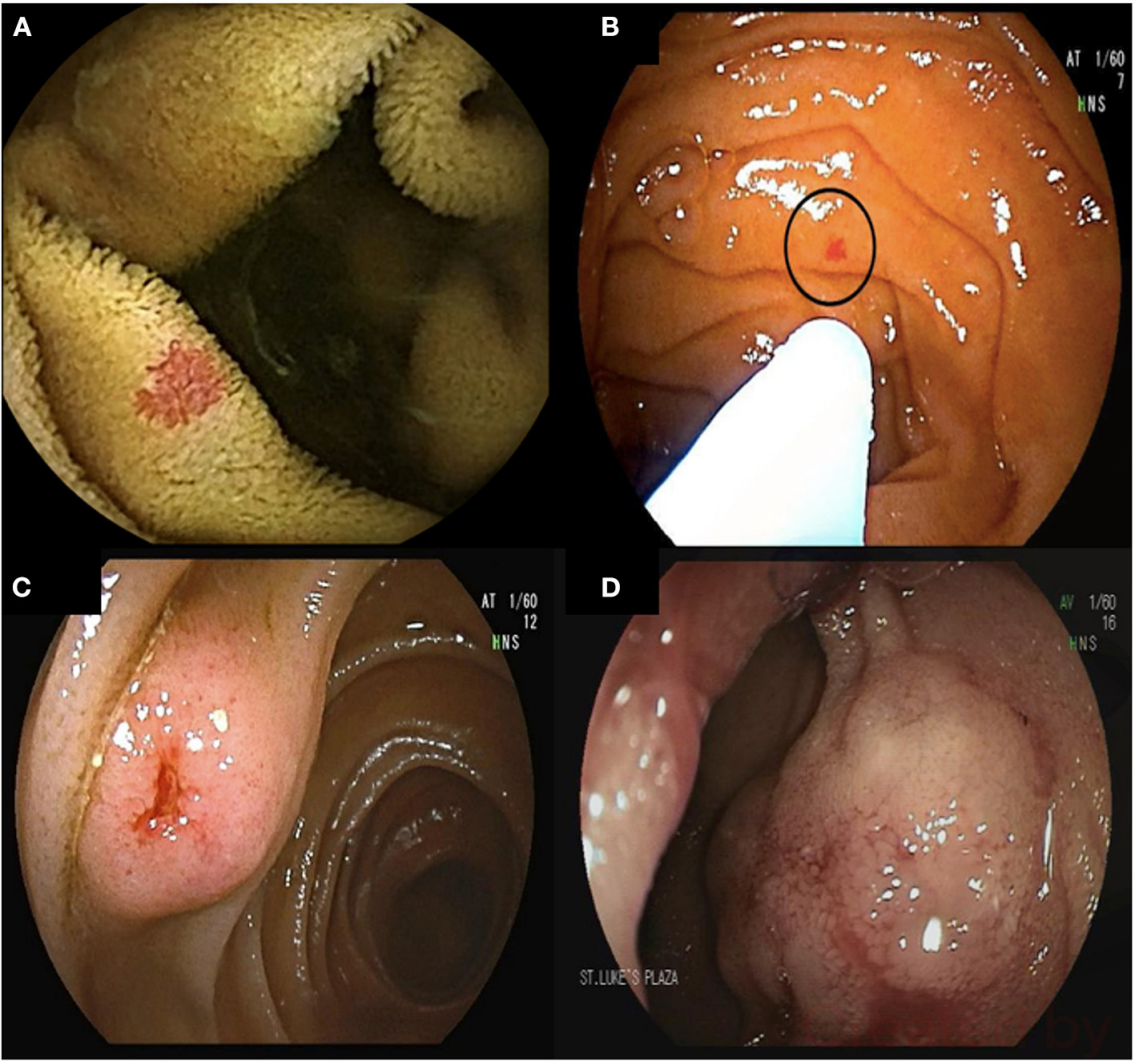
68-year-old male presented with melenic stools and a hemoglobin of 5.5 g/dL. Upper endoscopy and colonoscopy did not reveal the source of bleeding. Video capsule endoscopy revealed multiple proximal small bowel angioectasia **(A)**. Antegrade double balloon enteroscopy was performed with successful ablation of angioectasia using argon plasma coagulation **(B)**. Bleeding submucosal arteriovenous malformations (AVMs) found on deep enteroscopy requiring surgical resection **(C,D)**.

Small bowel bleeding remains the main indication for DAE and occurs in approximately 5% of patients presenting with GI hemorrhage (53, 54) (Figure 1).

The diagnostic yields of SBE and DBE in patients with small bowel bleed are similar ranging between 40–80% (21, 55, 56).

In a cost-effective study of patients with obscure GI bleeding, deep enteroscopy was the most cost-effective test after standard endoscopy for an endpoint of treatment or definitive diagnosis (57). Similarly, initial DE is a cost-effective approach for patients who likely have small bowel angiectasias (58). Initial VCE remains a common preferred strategy owing to its non-invasive nature.

Rebleeding rates for small bowel bleed after treatment during DBE were reported at 46% at 36 months in a large cohort of 261 patients. Risk factors for rebleeding include the total number of observed lesions and the presence of valvular or arrhythmic cardiac disease (59). May et al. showed a significant increase in hemoglobin levels and a decrease in blood transfusion requirements after therapy with argon plasma coagulation (APC) during DBE during a mean follow-up of 55 months (60). Other studies noted comparable rebleeding rates between patients with and without treatment of angiodysplasia (61).

### Small Bowel Tumors and Polyps

**Figure 2:**
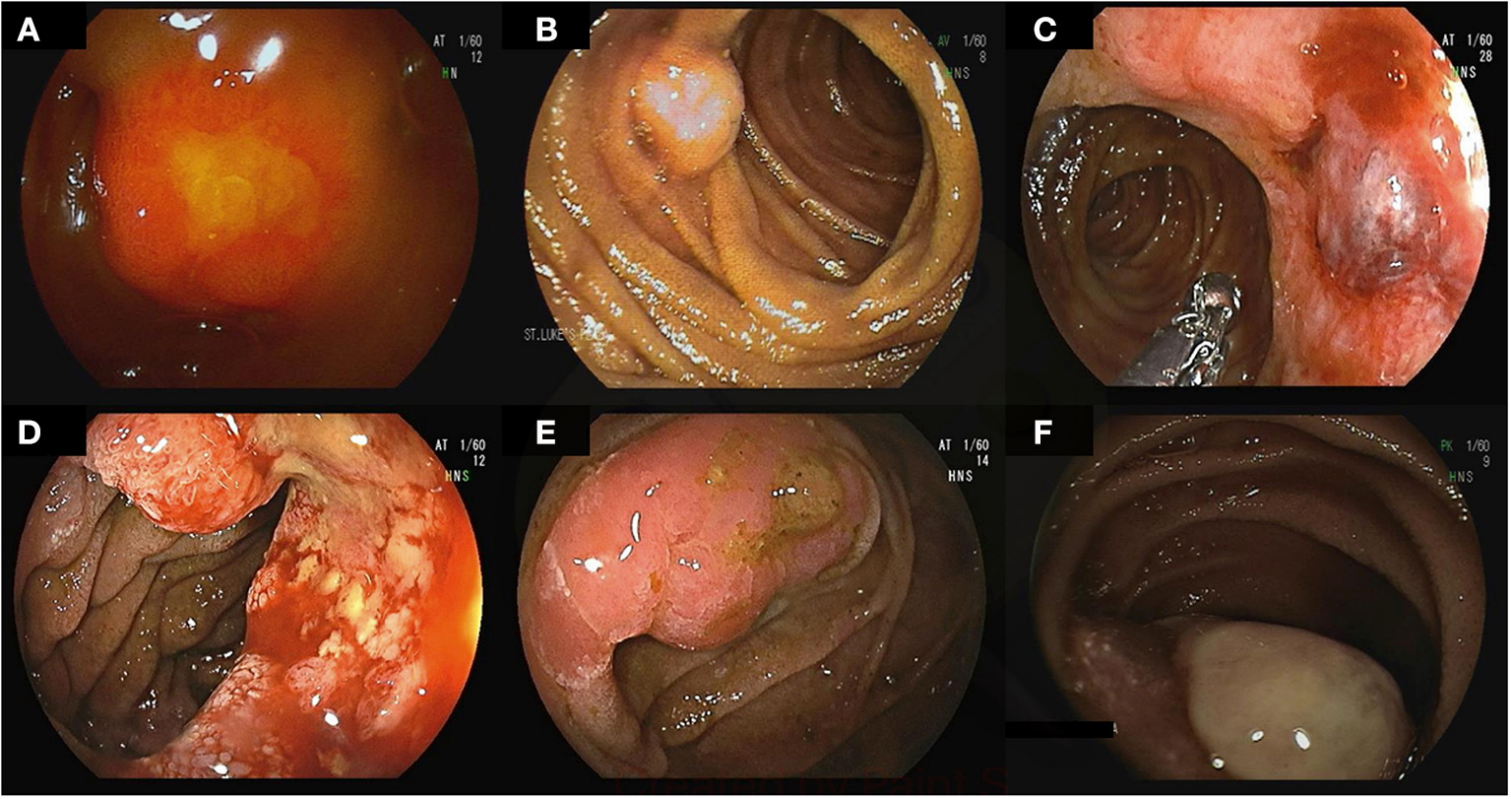
Small bowel tumors and polyps found on deep enteroscopy: well differentiated neuroendocrine tumors in the ileum **(A–C)**, moderately differentiated invasive adenocarcinoma in the jejunum **(D)**, tubulovillous adenoma with low-grade dysplasia **(E)**, small bowel metastasis secondary to renal cell carcinoma **(F)**.

Small bowel tumors account for 3–6% of all GI neoplasms (62). DAE techniques are effective in detecting and often treating small bowel tumors and polyps (Figure 2). The diagnostic yield for DBE in those with suspected small bowel pathology is between 9% to 14% (63–65). VCE was comparable to DBE in detection of small bowel tumors in a meta-analysis including 756 procedures (66). DE is also useful for patients in whom a suspicion for a small bowel tumor remains after a negative VCE. The reported miss-rate for small bowel tumors on VCE is 18.9% (67).

DE permits biopsy and tattoo placement to guide surgical resection in small bowel tumors. Endoscopic polypectomy has been reported in several studies without major complications. No differences were noted in the rates of therapeutic success between DBE and intraoperative enteroscopy, although the latter is much more invasive (68). Patients with polyposis syndromes can be managed endoscopically with DE decreasing the need for small bowel resections and short bowel syndrome (69).

### Crohn's Disease

**Figure 3:**
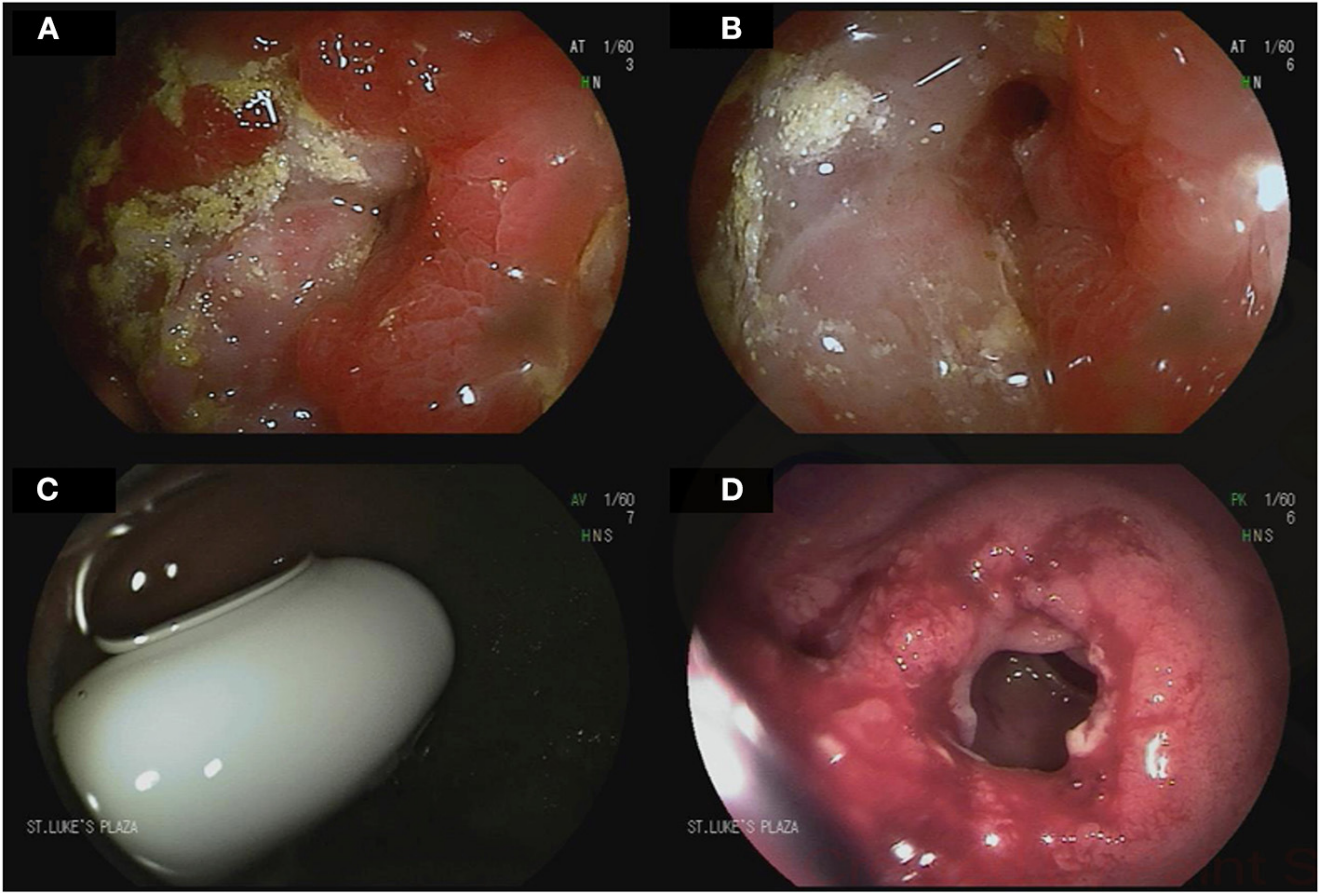
Device-assisted enteroscopy in the setting of stricturing small bowel Crohn's disease. A 70-year-old male with history of small bowel Crohn's disease on Infliximab was referred for deep enteroscopy after a small bowel follow through showed a stricture in the distal jejunum. Antegrade double balloon enteroscopy showed severe stenosis with friability and ulcerations **(A,B)**. Biopsies showed chronic enteritis with moderate activity. Biologic therapy for his Crohn's disease was adjusted accordingly. A 24-year-old male with small bowel Crohn's disease was referred for deep enteroscopy after retention of video capsule endoscopy in the small bowel. Retrograde double-balloon enteroscopy showed the capsule at the level of an ileal stricture **(C)**. The stricture was dilated using through-the-scope balloon dilation **(D)**.

DAE is less commonly used in Crohn's disease owing to its invasive nature, although Crohn's disease lesions are commonly found when DBE is performed (Figure 3) (70–72). It is mainly used for therapeutic interventions including balloon dilation of small bowel strictures and to obtain histological diagnosis in those with small bowel disease. In Crohn's disease patients with clinically suspected small bowel disease, 60% had active small bowel lesions on DBE leading in change in therapy in 75% of the cases (73). DBE-assisted small bowel stricture dilation can delay or prevent surgery with an acceptable complication rate (74).

## Indications for Device-Assisted Enteroscopy Outside of the Small Bowel

With improvements in deep enteroscopy, additional indications have emerged including DAE-assisted colonoscopy, endoscopic access to GI segments out of reach to conventional endoscopes, and ERCP in patients with altered anatomy.

### DAE-Assisted Colonoscopy

Overtube-assisted colonoscopy was shown to be useful in performing colonoscopy by increasing the cecal intubation rate and patient tolerance while decreasing the need for sedation (75). Cecal intubation rates were reported to exceed 90% in previous incomplete conventional colonoscopy (76). Single-balloon, double-balloon, and spiral enteroscopy were all reported to be effective and safe for this indication (77, 78). In addition, balloon overtube facilitates endoscopic submucosal dissection (ESD) by stabilizing the endoscope's position and improving maneuverability (79).

### DAE in Patients With Altered Anatomy

**Figure 4:**
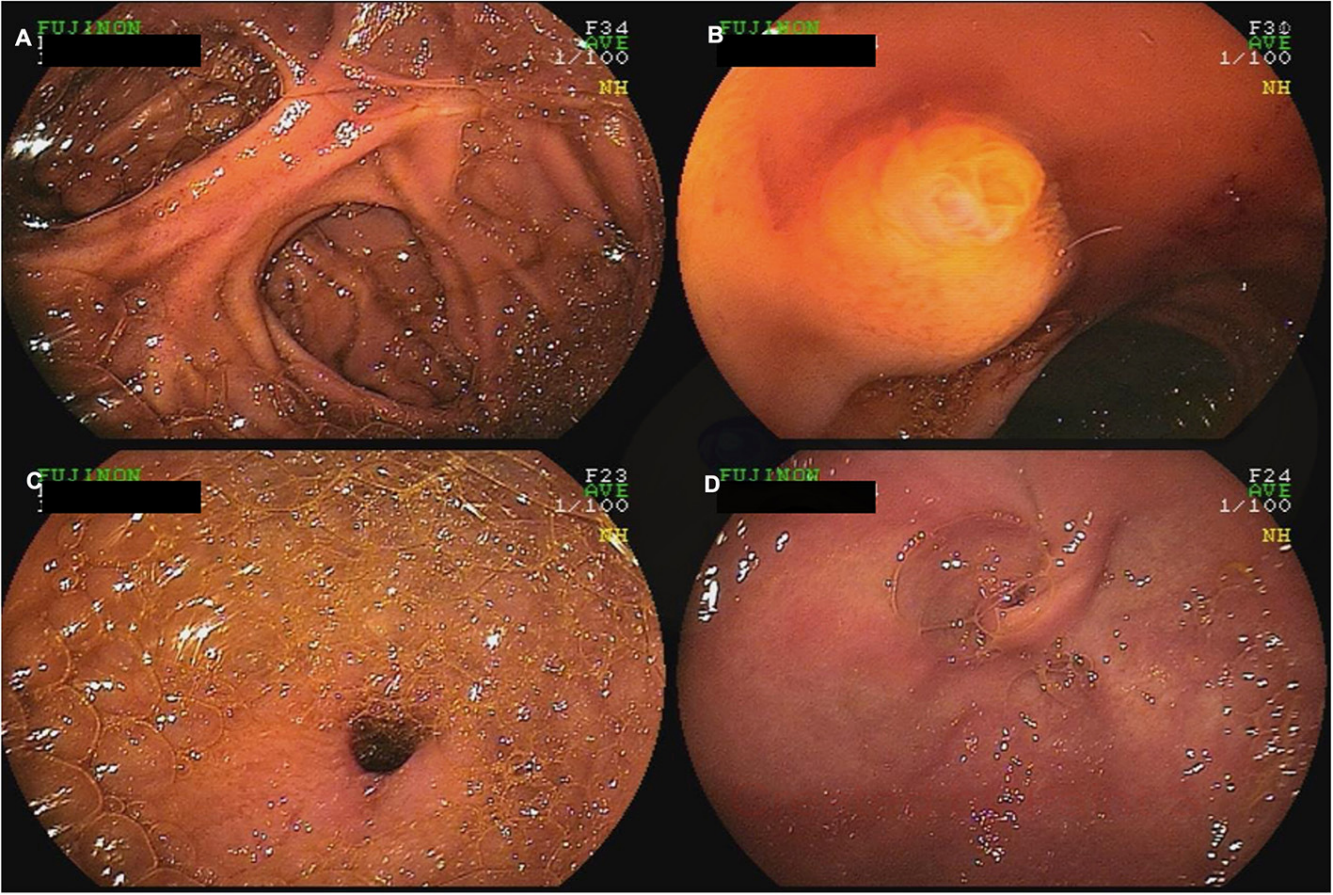
A 60-year-old female with a history of Roux-en-Y gastric bypass and persistent abdominal pain despite extensive work-up was referred for deep enteroscopy for evaluation of the gastric remnant. Antegrade double-balloon enteroscopy was performed showing the jejuno-jejunal anastomosis **(A)**, the major papilla **(B)**, the pylorus **(C)**, and the excluded stomach **(D)**.

DAE allows access to the excluded stomach in patients after Roux-en-Y gastric bypass allowing evaluation for bleeding and malignancy (Figure 4) (80, 81). Percutaneous endoscopic gastrostomy tube placement has also been described using DAE allowing permanent access to the upper gastrointestinal tract (82). Patients with intestinal surgical reconstruction can now benefit from DAE to evaluate or treat lesions out of reach to conventional endoscopes (83, 84). In particular, enteral insertion of self-expandable metal stents in intestinal segments previously excluded from endoscopic access has been described to treat malignant intestinal obstruction or strictures (85, 86). In addition, the newly developed shorter enteroscopes and the G-EYE enteroscopes allow through-the-scope deployment of enteral metal stents (83). However, DAE assisted enteroscopy in surgically altered anatomy is associated with an increased risk of small bowel perforation owing to adhesions (23).

### DAE-Assisted ERCP

Billroth II partial gastrectomy, Whipple's procedure, and Roux-en-Y anatomy are prone to an increased risk of biliopancreatic complications while rendering ERCP with a conventional side-viewing duodenoscope difficult (87). In a systematic review including 945 DAE-assisted ERCP in surgically altered anatomy, ERCP success was 74%, highest in patients with Billroth II and lowest in Roux-en-Y gastric bypass. The overall major adverse events was 3.4% (88). Given reported technical challenges with DAE-assisted ERCP using conventional double or single-balloon enteroscopy, shorter DAE endoscopes were developed allowing the use of conventional ERCP accessories and stents and an additional water channel allowed flushing away biliary stones and blood without the need to clear the working channel (89). Enteroscopes with a working-channel of 3.2 mm currently allow biliary self-expandable metal stent insertion which was impossible until recently (90). The 200-cm long DAE may be particularly helpful with Roux-en-Y bypass with a long limb. Forward viewing enteroscopes also facilitate direct cholangioscopy in patients with altered anatomy allowing introduction of the enteroscope into the biliary system after balloon dilation of the papilla followed intraductal endoscopic procedures such as biopsy sampling and stone extraction (91, 92). The use of a plastic cap at the tip of the enteroscope may facilitate cannulation of the papilla (93). CO2 insufflation is also recommended all cases of therapeutic endoscopy including DAE-assisted ERCP. PowerSpiral Enteroscopy-ERCP has also been described in Roux-en-Y anatomy. The speed, depth and control of insertion, short length of 168 cm, and 3.2-mm working channel offer potential advantages compared to standard DAE (94).

## Future Directions

DAE is continuously evolving with new and improved enteroscopes allowing more complex therapeutic endoscopy procedures. PSE appears to be a promising and exciting advancement in deep enteroscopy. It may be the solution to finally assess the small bowel completely, reliably, and with relative speed all in one setting. Future randomized controlled trials will be needed to assess its ultimate benefit. PSE may be the start of an endoscopic motorized revolution that opens the world of endoscopic technology in many areas.

In the past few years, deep learning has revolutionized the field of computer vision and an increasing number of studies utilizing artificial intelligence in VCE has been published. Deep learning has achieved excellent sensitivity and specificity in detection of small bowel diseases (95). Eventually, this will translate to DAE by improving its diagnostic yield and performance. In addition, the implementation of robotics in flexible endoscopy appears to provide greater stability and controllability for complex therapeutic procedures that may eventually be applied to deep enteroscopy further expanding its therapeutic armamentarium (96).

## Conclusion

DAE is becoming a standard tool in the evaluation and management of small bowel diseases. Particularly, DBE and SAE have proven their value and safety in large cohort studies (Table 2). The introduction of PSE may represent a major advance in small bowel endoscopy if efficacy and safety results can be replicated in larger studies. Although capsule endoscopy will remain the initial diagnostic test in most patients with suspected small bowel diseases, the future of deep enteroscopy appears promising given the efficacy, simplicity, and safety of motorized spiral enteroscopy.

**Table 2:** Characteristics of currently available enteroscopy techniques.

	**Company**	**Depth of maximal insertion, antergrade**	**Diagnostic yield**	**Total enteroscopy rate**	**Average procedure time, antegrade (minutes)**	**Major complication rate (%) (includes perforation, pancreatitis, bleeding)**	**Advantages**	**Disadvantages**
**Push Enteroscopy**		60–80 cm	15–40%	0%	30	0.1–0.3	- Shortest sedation and procedure time - Wide availability and ease of use	- Evaluation limited to proximal jejunum
**Double Balloon Enteroscopy**	Fujifilm, Tokyo, Japan	220–360 cm	40–80%	40–60%	60–123	0.72–1.2	- Higher depth of insertion and total enteroscopy rate compared to SBE - Most studied technique in safety and efficacy	- Lengthy procedure time - Longer time to achieve competency - Two operators required
**Single Balloon Enteroscopy**	Olympus, Tokyo, Japan	133–270 cm	41–65%	15–25%	57–72	0.02	- Shorter procedure time and easier use compared to DBE	- Lower depth of insertion and total enteroscopy rate compared to DBE
**Balloon Guided Endoscopy**	NaviAid, Smart Medical Systems, Israel	120–190 cm	45–59%	N/A	15–52	Limited data	- No special preloading and preparation needed - Device inserted *via* instrument channel as needed	- Very limited data on efficacy and safety
**Manual Spiral Enteroscopy**	Spirus Medical, Stoughton, Massachusetts	175–262 cm	30–65%	10%	35–52	0.08	- Shorter procedure time compared to balloon assisted enteroscopy	- Difficult retrograde passage - Low total enteroscopy rate - Two operators required
**Motorized Spiral enteroscopy**	Olympus, Tokyo, Japan	450–490 cm	65–80%	60–70%	40	1.5	- Includes large 3.2 mm accessory channel and a separate irrigation channel - Short procedure time and easy to use - Highest total enteroscopy rate	Limited data on safety - Prophylactic esophageal dilation may be required - Limited availability

*SBE, single balloon enteroscopy, DBE, double balloon enteroscopy*.

## Author Contributions

RC, HG, and AP: conception and design. FN and HG: first draft. All authors: critical revision and editing, and final approval.

## Conflict of Interest

The authors declare that the research was conducted in the absence of any commercial or financial relationships that could be construed as a potential conflict of interest.

## Publisher's Note

All claims expressed in this article are solely those of the authors and do not necessarily represent those of their affiliated organizations, or those of the publisher, the editors and the reviewers. Any product that may be evaluated in this article, or claim that may be made by its manufacturer, is not guaranteed or endorsed by the publisher.
